# Adherence to Report and Patient Perception of an Interactive App for Managing Symptoms During Radiotherapy for Prostate Cancer: Descriptive Study of Logged and Interview Data

**DOI:** 10.2196/cancer.7599

**Published:** 2017-10-31

**Authors:** Ann Langius-Eklöf, Mats Christiansen, Veronica Lindström, Karin Blomberg, Maria Hälleberg Nyman, Yvonne Wengström, Kay Sundberg

**Affiliations:** ^1^ Division of Nursing Department of Neurobiology, Care Sciences and Society Karolinska Institutet Huddinge Sweden; ^2^ School of Health Sciences Örebro University Örebro Sweden

**Keywords:** mobile apps, mHealth, prostate cancer, symptom assessment

## Abstract

**Background:**

Patients undergoing radiotherapy for prostate cancer experience symptoms related to both the cancer itself and its treatment, and it is evident that patients with prostate cancer have unmet supportive care needs related to their disease. Over the past decade, there has been an increase in the amount of research within the field of mobile health and the use of apps as tools for managing illness. The main challenge is to develop a mobile technology to its full potential of being interactive in real time. The interactive app Interaktor, which aims to identify and manage symptoms in real time includes (1) a function for patients’ assessment of the occurrence, frequency, and distress of symptoms; (2) a connection to a monitoring Web interface; (3) a risk assessment model that sends alerts via text message to health care providers; (4) continuous access to evidence-based self-care advice and links to relevant websites for more information; and (5) graphs for the patients and health care providers to view the history of symptom reporting.

**Objective:**

The aim of the study was to investigate user behavior, adherence to reporting, and the patients’ experiences of using Interaktor during radiotherapy for localized advanced prostate cancer.

**Methods:**

The patients were instructed to report daily during the time of treatment and then for an additional 3 weeks. Logged data from patients’ use of the app were analyzed with descriptive statistics. Interview data about experiences of using the app were analyzed with content analysis.

**Results:**

A total of 66 patients participated in the study. Logged data showed that adherence to daily reporting of symptoms was high (87%). The patients used all the symptoms included in the app. Of the reports, 15.6% generated alerts to the health care providers. Overall, the patients found that it was easy and not particularly time-consuming to send a daily report, and many described it as becoming a routine. Reporting symptoms facilitated reflection on their symptoms and gave them a sense of security. Few technological problems were reported.

**Conclusions:**

The use of Interaktor increased patients’ sense of security and their reflections on their own well-being and thereby served as a supportive tool for the self-management of symptoms during treatment of prostate cancer. Some further development of the app’s content might be beneficial for future use.

## Introduction

### Background

Prostate cancer is the most common form of cancer in men and occurs mainly in middle and older age [1]. Depending on disease stage, patients are offered three alternative options, that is, expectation, surgery, (prostatectomy) or radiotherapy for 5 to 8 weeks [2]. Overall, there is evidence that patients with prostate cancer have unmet supportive care needs during and after treatment, as well as when they are under long-term surveillance [3]. These needs are multifocal, and they relate to physical, emotional, social, and intimacy needs and vary over time and between treatment modalities. During radiotherapy, patients with prostate cancer experience symptoms related to both the disease and the treatment, for instance, urinary symptoms, bowel symptoms, pain, and fatigue [4-6]. Patients report using different strategies to alleviate symptom burden with a variation in outcomes [7,8]. Furthermore, self-care advice from clinicians for managing symptoms during radiotherapy varies greatly in both quantity and content [4]. There is limited evidence on how to design interventions for managing symptoms [9] despite the acknowledgment that undiagnosed symptoms impact the quality of life and recovery of patients with cancer [10]. It is proposed that care and support for patients with cancer should include early recognition of signs and symptoms, support for self-care, personalized care planning, and routine use of patient-reported outcome measures (PROMs) [11]. Routine use of PROMs in cancer care seems to facilitate the identification of present problems and impact of treatment, and enhances patient-clinician communication that promotes shared decision making [12,13]. There are some promising studies that have used Web-based PROMs with interactive components to support patients with cancer to deal with their disease by monitoring symptoms, providing self-care advice, and giving access to clinicians [14-16]. Ruland et al [14] used a Web-based system that included components for patients’ assessment of symptoms, provision of triggered self-management support, e-communication with expert cancer nurses, an e-forum with other patients, and access to a diary for personal notes. Furthermore, in the randomized controlled study including patients with breast and prostate cancer, there was a slight favor in the intervention group on overall symptom distress [14]. In another study, a Web-based interface for reporting symptoms related to chemotherapy was tested [15]. Patients randomized to use the Web interface before each visit to the oncology clinic showed considerable improvement in the quality of life and had fewer emergency visits and remained longer on chemotherapy than those patients receiving usual care. Another study showed that weekly Web-mediated follow-up of self-reported symptoms in a group of patients with advanced lung cancer improved overall survival in comparison with patients having routine follow-up [16]. During the last decade, there has been an increasing interest within the field of mobile health (mHealth), which has shifted from focusing on the technical development to how the use of apps can influence people and their health [17]. A review of how mHealth is used in different phases of cancer treatment revealed that most reports focus on support in medical decision making and much less on how to support patients during the entire care process [18]. A mobile phone–based remote monitoring system for real-time collection of PROMs aiming to provide structured self-care has proven to be feasible and acceptable for use by the patients but not developed for prostate cancer [19]. Paterson et al [20] tested a real-time electronic diary for prostate cancer survivors and showed high response rate and acceptability among the patients.

More studies concerning the use of apps are warranted as it is still in its initial phase [21] and its full potential is not used regarding evidence-based content, usability, security, and interactivity [22,23].

In collaboration with a Swedish company, Health Navigator, that specializes in health care management and new innovative care solutions, an interactive app (Interaktor), for smartphones and tablet computers has been developed. The theoretical underpinning in the developmental process was person-centered care [24]. In person-centered care, the importance of integrating the patients’ perspective in the care process and attaining interaction between the patient and the care provider is emphasized. It is essential to enable patients to actively participate in their care rather than being passive receivers of care [25]. Interaktor includes (1) a function that allows patients’ assessments of the occurrence, frequency, and distress of symptoms, which are immediately available to health care providers; (2) a connection to a monitoring Web interface and logged data storage on a secure server; (3) a risk assessment model for symptoms of concern that sends alerts via text message to the health care providers; (4) continuous access to evidence-based self-care advice related to reported symptoms and links to relevant websites for more information; and (5) graphs for the patients and health care providers to view the history of symptom reporting. Interaktor is generic and can be adjusted for different diagnoses and settings. The content of each version is developed in partnership with patients and health care professionals and by reviewing the contemporary literature.

### Objectives

This study involves a prostate cancer version for use during radiotherapy. The radiotherapy is predominantly given at outpatient clinics, which means that the patients largely manage their symptoms and concerns at home based on information and advice provided by the clinic. There is a clear knowledge gap on how to support patients with prostate cancer in an effective and timely manner during radiotherapy. Therefore, testing Interaktor during treatment in outpatient care was considered appropriate to identify its potential to be beneficial for easing symptom burden.

In previous feasibility studies, the version of Interaktor for prostate cancer and a version for older adults with homecare were observed to be acceptable and user-friendly [26,27]. Patients with prostate cancer using Interaktor during radiotherapy reported reduced symptom burden compared with those who did not use the app [28]. However, it is important to also assess the patients’ experiences with using a new technology [29]. Therefore, the aim of this study was to investigate user behavior, adherence to reporting, and experiences of using Interaktor during radiotherapy for localized prostate cancer.

## Methods

### Study Design and Recruitment

The study was conducted at two university hospitals, one urban and one rural, where the intervention group that used Interaktor during radiotherapy was compared with a historical control group [28]. This study comprises logged data and interviews with patients in the intervention group. Patients scheduled for radiotherapy of prostate cancer at the two clinics were consecutively invited to participate in the study. The inclusion criteria were locally advanced prostate cancer planned for radiotherapy and being literate in Swedish and physically, psychologically, and cognitively able to participate in the study assessed in a conversation between the researchers and the patients. The intention of treatment was curative. Treatment was administered according to the national guidelines [30], including either external beam radiation therapy (EBRT) for 5 weeks or EBRT with a combination of iridium high-dose-rate brachytherapy for 8 weeks both with adjuvant hormone therapy based on tumor stage. Depending on the regimen, the patients had the ability to report between 56 and 77 days.

### Description of the Prostate Cancer Version of Interaktor

This version includes 14 identified [4] and tested [26] symptom questions regarding bladder (urinary urgency, difficulties in urinating, urinary leakage, and hematuria) and bowel (diarrhea, stool leakage, obstipation, and blood in stool) function, fatigue, pain, worry, depression, sleep, and flushing. There is also an open comment section—Other symptoms or concerns to report—that provides opportunity to the patients to add comments. Patients are asked about the symptoms’ occurrence, frequency, and the distress level based on a structure used in a standardized symptom and quality of life questionnaire [31] (eg, Do you experience urinary urgency?, and if the answer is yes, the patient is asked how often—never, sometimes, rather often, or very often—followed by how distressing—not at all, a little, rather, or very much—the symptom is). The symptoms of insomnia, obstipation, and blood in the stool are only assessed by the distress level because these symptoms are not appropriate to report regarding frequency on a daily basis.

Some of the reported symptoms generate an alert, defined by symptom frequency or distress level, to registered nurses at the respective clinics. The levels at which alerts are triggered are the same for all patients and are set according to a risk assessment model based on consultations with health care professionals caring for this group of patients. The conclusion was to differentiate symptoms into alerts that demand rather instant care (such as a prescription for a painkiller or a coaching conversation), or that represent an acute threat to the patients’ health and are a direct cause of seeking emergency care if left unattended for too long. The alerts were set regarding urinary urgency, difficulties in urinating, obstipation, blood in stool, pain, worry, depression, and hematuria. There are two kinds of alerts—yellow alerts that request a nurse to contact the patient during the same day, for example, reporting having pain sometimes, and red alerts requiring contact within 1 hour, for example, reporting urinating difficulties as often or almost always.

**Figure 1:**
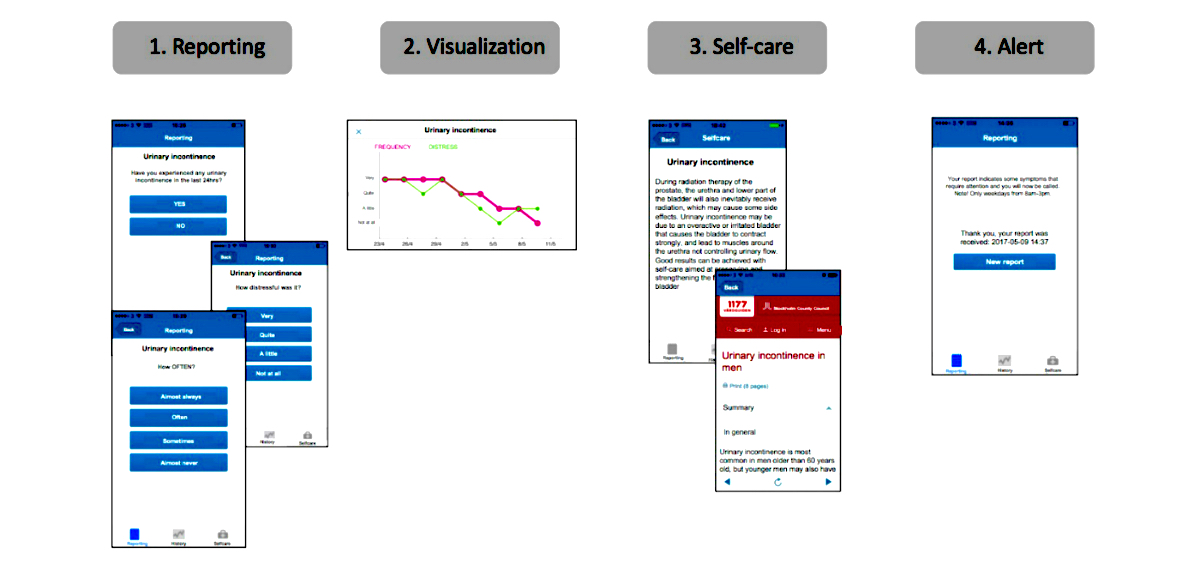
Illustration of the Interaktor app.

A total of 16 self-care advice regarding symptoms related to prostate cancer and radiotherapy are included in the app together with relevant links to evidence-based Web pages. An overview of the components in Interaktor is presented in Figure 1.

### Study Procedure

All of the patients were provided with a smartphone belonging to the project with the app Interaktor installed and were requested to report their symptoms daily (or more often if they wished) during office hours on weekdays throughout the radiotherapy period and for 3 weeks after. The patients were given thorough instructions by the researchers on how to use the app and a written checklist including a phone number for technical support. The patients were given an individual log-in and personal identification number (PIN) to get access to the app. They were also informed that in case of an alert, a nurse would call them during office hours on their home phone number until they could be reached. Any acute problem occurring at other time points had to be handled according to the standard procedure of the oncological clinic, that is, a certain phone number at the clinic to call for advice. A notification was sent out as a reminder to the patients to make a report in the app if they had not reported by 3 PM. The patient’s self-report was sent directly via the secure server and was accessible from a Web interface for the nurses at the hospitals and the researchers at the university. The average time required for reporting was estimated to be 5 minutes [26].

### Data Collection and Analysis

Data were collected from two sources, which are (1) logged data from database, and (2) telephone and face-to-face interview.

First, logged data extracted from the database, which included (1) the total number of reports, (2) the number of reports per symptom, (3) the severity and distress levels of symptoms, (4) the alerts generated, (5) patients’ responses to the open-ended question, and (6) actions on alerts. The readings of self-care advice and historical graphs were not logged.

Second, data obtained from telephone and face-to-face interviews conducted by 3 members of the research team shortly after the end of using the app. The interviews followed a semistructured guide with the initial question “What was it like to report in the mobile phone?” (Table 1). The interviews lasted for 10 to 15 minutes, and during the interviews, the researchers wrote down the answers as close to verbatim as possible in a template following the interview guide.

**Table 1:** The semistructured interview guide.

Question		Follow-up question
1.	What was it like to report in the mobile phone?	Difficulties and benefits or opportunities?
2.	How did you experience the technology?	
3.	How did you perceive the questions?	Relevant or something missing?
4.	What was it like to report daily?	
5.	Was it relevant to report from the beginning of treatment to 3 weeks after the end of treatment?	
6.	Have you been contacted by a nurse after an alert?	If so, your experience?
7.	Can you describe what use you have had of the self-care advice?	
8.	Can you describe how you used the Internet links?	
9.	Can you describe how you used the historical graph?	
10.	Is there anything else you want to add?	

Logged data were analyzed using descriptive statistics. The statistical procedures were performed in Microsoft Office Excel 2010 (Microsoft Corporation, Redmond, WA, USA) and IBM SPSS (version 23.0 for Windows, Chicago, IL, USA). The logged symptom data were organized by frequency (1=very seldom, 2=sometimes, 3=often, or 4=almost always) and by how distressing the symptom was (1=not at all, 2=a little, 3=rather much, or 4=very much).

The analysis of the notes taken during the semistructured interviews was conducted using summative content analysis [32]. The verbatim notes from the patient interviews were read through by 2 of the authors to gain familiarity with the content. Subsequently, the authors independently identified codes that responded to the study aim. The codes were discussed between the 2 authors regarding differences and similarities and how well they covered the content of the interviews. The harmonized codes were transferred to an Excel spreadsheet, whereas their relationships were identified for organizing them into categories. The codes and categories were discussed and verified with the other authors. A quantification within the categories was also performed to visualize potential patterns [33,34].

### Ethical Aspects

Ethical approval for the study was obtained from the Regional Ethical Review Board of Uppsala University (dnr 2011/256). All participants gave their oral and written consent to participate. This study was designed to meet the ethical principles for research described by the International Council of Nurses by ensuring anonymity, integrity, and confidentiality for the participants [35]. To assure that all participants had equal access and ability to participate in the study, participants were lent a smartphone.

## Results

### Enrollment and Sample Characteristics

There were 107 eligible patients in the intervention group, but 34 patients declined or could not be reached and 7 did not fulfill the inclusion criteria leaving 66 ( 61.7%) patients that participated in the study. The patients’ mean age was 69 years, and further clinical and demographic data are presented in Table 2.

A total of 53 patients participated in the interviews (face-to-face, n=9) regarding the experience of using Interaktor. There were 13 patients that did not answer repeated telephone calls from the researcher after they finished reporting through the app.

### Logged Data

A total of 3 patients filled out the report once, when instructed about the app, but did not file any further reports during the study period. The logged data from the remaining 63 patients showed that adherence to reporting symptoms daily was on average 87% (median 92%, range 16%-100%). The patients had in total sent in 3536 reports during the study period, and the patients reported 10,025 specific symptoms in total. All of the symptoms included in the app were used by the patients (Table 3). The most common symptoms reported were urinary urgency (18.70%), fatigue (18.33%), hot flushes (16.17%), and difficulties in urinating (10.50%).

Of the 10,025 reported symptoms, 1566 (15.60%) generated alerts to the nurse at the oncology clinic (Table 4). Out of these alerts, 517 (33.00%) alerts were considered severe (red), and 1049 (67.00%) were considered less severe (yellow). The alerts were most commonly related to urinary urgency (yellow n=359, red n=127), pain (yellow n=287, red n=212), and difficulties in urinating (yellow n=274, red n=72). All of the alerts led to the nurses contacting the patients and adding a written note in the system such as “Telephone call to the patient – no further action,” “Pain same as before – already been taken care of,” “Extension of the patient’s prescription,” “Booked an appointment with the physician,” and “Advice given on the patient’s medication.”

**Table 2:** Clinical and sociodemographic characteristics of the study participants.

Clinical and sociodemographic characteristics		Descriptive analyses (N=66)
**Age, years**		
	Mean (SD)	69 (5.8)
	Median (range)	70 (53-82)
**Living situation, n (%)**		
	Married or living with partner	57 (86)
	Living alone	9 (14)
**Education level, n (%)**		
	Junior compulsory	9 (14)
	Senior high school	23 (36)
	Postgraduate or university	32 (50)
**Occupation, n (%)**		
	Working, retired	59 (89)
	Sick leave	7 (11)
**Clinical tumor stage, n (%)**		
	1	16 (24)
	2	29 (44)
	3	17 (26)
	Missing	4 (6)
**Treatment, n (%)**		
	Adjuvant hormonal therapy	50 (76)
	EBRT^a^	20 (30)
	Brachytherapy combined with EBRT	46 (70)

^a^EBRT: external beam radiotherapy.

**Table 3:** Occurrences, frequency, and distress of the symptoms as reported in the app by patients with prostate cancer (N=63) during their radiotherapy.

Symptoms (number of patients reporting at least once)	Occurrence	Frequency		Distress (N=14)	
	n (%)	Mean (SD)	Range	Mean (SD)	Range
Urinary urgency (n=60)	1875 (18.70)	2.18 (0.676)	1-4	2.07 (0.437)	1-3
Fatigue (n=58)	1838 (18.33)	2.22 (0.589)	1-4	2.06 (0.577)	1-4
Hot flushes (n=43)	1621 (16.17)	1.99 (0.483)	1-4	1.89 (0.429)	1-4
Difficulties in urinating (n=47)	1053 (10.50)	2.28 (0.692)	1-4	2.22 (0.734)	1-4
Pain (n=46)	685 (6.83)	2.30 (0.714)	1-4	2.38 (0.559)	1-4
Insomnia (n=43)	651 (6.49)	N/A^a^	N/A	2.20 (0.592)	1-4
Diarrhea (n=48)	598 (5.97)	2.08 (0.525)	1-4	2.10 (0.536)	1-4
Urinary leakage (n=27)	358 (3.58)	1.94 (0.568)	1-3	2.14 (0.602)	1-4
Stool leakage (n=29)	273 (2.72)	1.59 (0.527)	1-3	2.01 (0.756)	1-4
Obstipation (n=30)	255 (2.54)	N/A	N/A	2.20 (0.689)	1-4
Depression (n=28)	253 (2.52)	2.29 (0.885)	1-4	2.39 (0.780)	1-4
Worry (n=23)	248 (2.48)	1.95 (0.724)	1-4	2.20 (0.610)	1-4
Hematuria (n=33)	175 (1.75)	2.23 (0.833)	1-4	1.79 (0.497)	1-3
Blood in stool (n=22)	142 (1.42)	N/A	N/A	1.93 (0.608)	1-4

^a^N/A: not applicable.

**Table 4:** Distribution of the alerts as reported in the app by patients with prostate cancer (N=63) during their radiotherapy presented on symptom and alert levels.

Symptoms (number of patients reporting)	Yellow alerts, N=1049, n (%)	Red alerts, N=517, n (%)
Urinary urgency (n=52)	359 (34.22)	127 (24.6)
Pain (n=63)	287 (27.36)	212 (41.0)
Difficulties urinating (n=44)	274 (26.12)	72 (13.9)
Depressed (n=13)	75 (7.15)	38 (7.4)
Worry (n=16)	29 (2.77)	36 (6.9)
Hematuria (n=21)	25 (2.38)	32 (6.2)
Obstipation	0 (0.00)	0 (0.0)
Blood in stool	0 (0.00)	0 (0.0)

A total of 47 (75%) patients sent 433 free-text comments through the open question. These mainly consisted of the message “You don´t need to call, my symptom is the same as yesterday.” Other free-text messages were such as “I have back pain but cannot see how this could be related to the treatment” or reporting another symptom not included in the app, for example, “I feel dizzy.” The free-text was also used for other communications with the nurses such as wishing the nurse a good weekend or describing upcoming plans for the patient’s weekend.

### Patients’ Perceptions of the App

The analysis of the interviews resulted in the following six categories: reporting and content, self-care advice, historical graphs, alerts, technology, and safety and novelty. Overall, the patients reported that it was easy to use the app, even those few who were not accustomed to smartphones. It was not particularly time-consuming to send reports daily, and the patients described reporting as becoming a routine. Reporting symptoms was described as making the patients reflect over their own well-being.

### Reporting and Content

According to the patients (n=44), the possibility to report daily facilitated reflection on their symptoms and illness:

When I answered the questions, I thought a lot about how I was feeling...It gave me perspective on my illness...I was feeling pretty good after all...

P6, age 73 years

The content and the design of the questions were described as relevant by the majority of the patients (n=48); however, some (n=10) said that it was sometimes difficult to nuance the answer alternatives:

Relevant questions, but might be a little blunt; hard to know what is meaningful to report, hard to put the level of how to respond to such as “not at all” or “a little” distress in the beginning.

P58, age 74 years

Some patients (n=16) wanted the possibility to say more about the symptoms, and 3 said that the app lacked symptoms such as gas in the stomach and dizziness.

The reporting sometimes became a routine for the patients (n=20) commenting that:

I did it every morning after listening to the news on the radio.

P56, age 72 years

Some patients (n=7) said that they appreciated the reminder that came at 3 PM, if they had not submitted a report earlier that day.

### Self-Care Advice

The self-care advice was read by the majority of patients (n=43). Many of them (n=25) reported that the advice had been important to them, particularly concerning knowledge (n=18) and support to alleviate symptom burden (n=7). A few patients (n=5) said that they had already received the information from the nurses about self-care advice or side effects, or they had decided that they did not need or want to use that feature of the app.

### Historical Graphs

A total of 21 patients reported that they followed their symptoms over time in the graphs, and they described how this function gave them and their families confirmation of their well-being:

I looked at the lines and it gave me in some strange way a confirmation of how I was feeling

P24, age 69 years

I used the graphs to show my family and friends that I actually felt good during the treatment.

P21, age 73 years

Some patients (n=13) stated that they did not follow their own graphs, mainly because they had forgotten they had the option to do so (n=7).

### Alerts

To be contacted by the nurses in connection with an alert was described as positive (n=19) through the direct dialogue with the nurses:

It felt good to be called by the nurse... it was a confirmation that it worked...I felt like a VIP and my problem was easily solved by just talking to the nurse.

P21, age 73 years

There were also patients who expressed a wish to decide for themselves when to call the nurse (n=10). A total of 4 patients did not want to be contacted because of alerts, and they described how they had learned to adjust their responses to avoid a call from the nurse:

It took me about a week to fine tune the level at which to report symptoms. At the start the nurses called me pretty often, but then I learned how to report the symptoms so as to avoid being contacted unnecessarily.

P29, age 55 years

### Technology

The majority of the patients (n=37) had not experienced any technological problem commenting that:

There was no problem at all with the phone...not at al...it was so easy to use that anyone can learn to use it...even for me as a non-technical person...

P16, age 72 years

The technological problems that were reported by the patients were primarily connected to the beginning of the reporting period (n=20). Technological problems such as sending the report and having problems moving on to the next question in the app were solved by the patients themselves by restarting the smartphone. Other technical issues described by the patients were related to the server (n=2), insufficient connection to the network (n=3), and the need to log-in with the PIN each time they reported (n=2).

### Safety and Novelty

Several patients stated that the app gave a sense of security (n=21) in the form of being seen, monitored, and prioritized by the health care providers:

It felt like it was easy to get in touch with a nurse who was online all the time, it has felt really good.

P64, age 76 years

Some patients (n=8) described the novelty of the app for future patients and how it could be of support to both patients and staff:

It almost feels like having health care staff in one’s home. I think there may be some kind of...perhaps less burden on the health care.

P10, age 75 years

A few patients (n=3) brought up a sense of lack of safety mainly related to an alert that did not result in contact from the nurse, which made them question the technology:

I was disappointed when no one called...it seems questionable whether the system can be trusted.

P59, age 72 years

## Discussion

### Principal Findings

This study shows high adherence to the daily reporting of symptoms through an interactive smartphone app (Interaktor) among a group of patients with prostate cancer during the entire period of radiotherapy and 3 weeks afterwards. In Borosund et al’s [36] study, 64% of the patients having access to their Web-based system (described in the Introduction above) for 1 year logged in twice or more. There were no significant differences between users and nonusers but a trend of higher use among patients with prostate cancer, no comorbidity, and more computer experience. In Basch et al’s study [15], the attrition rate was 73% in completing a Web-based self-report. Furthermore, patients with prostate cancer have shown high attrition rates in filling in a daily electronic diary [20]. Hence, there should be no reason to hinder further implementation of mHealth based on the argument of fear of technology. The patients in this study did not find reporting symptoms every day to be burdensome; on the contrary, they appreciated it as it gave them a sense of security even when being at home and not in a hospital or clinic. This is in contrast to a study that reported that older adults found it intrusive to be asked about their illness on a daily basis [29]. All symptoms included in Interaktor were used during the study period, and the symptoms were relevant to the patients. Altogether, 16% of the reported symptoms generated an alert to the nurses, which confirms the literature that patients with prostate cancer may have severe symptoms during radiotherapy [4-6]. There were numerous yellow alerts for pain and problems with urinating; symptoms not necessarily perceived by the patients as distressing enough to generate an alert. Another indication that the level was set too low for some alerts is that some patients described how they learned to fine-tune their responses to avoid being contacted by the nurses. This suggests that the risk assessment model should be refined in a future study or before implementation of the app in the clinic. Furthermore, 3 alerts out of 1500 alerts did not lead to any call from the nurse. However, the reason whether this was a technical error or a human error cannot be ascertained because this was reported ex post facto in the posttrial telephone interviews. Overall, only a few patients reported technological problems, and those problems mainly related to problems connecting to the server and the Internet and the need to log in with a PIN code every time. However, it is important to be aware that there is the risk of false reassurance if the technology fails [37]. This stresses the importance of the technology and operation services being optimized and maintained. The reading of self-care advice and viewing of graphs could not be logged in this study, something that should be considered for future development of the app. The majority of the patients stated in the interviews that they read the self-care advice or followed their symptom history in the graphs, and they reported it as supportive. Borosund et al [36] found that the patients’ use of all of the components in their Web-based system was related to low social support and high levels of depression in the group of patients with breast cancer but not in the group of patients with prostate cancer [36]. Whether these results relate to gender or cancer diagnosis cannot be concluded. Overall, the patients in this study appreciated the use of Interaktor and expressed feelings of being secure, which has been described before but in a smaller study [26].

It was hypothesized that Interaktor should enhance patients’ participation in their own health care and that taking an active role will lead to better well-being and health. The theoretical underpinning (based on person-centered care [25]) in the development of Interaktor was to consider that patients have different needs when managing symptoms and concerns in connection with an illness. The results showed that the patients used Interaktor in different manner in line with the intention. Almost all patients reported daily, some used the graphs for their own symptom monitoring, some used the self-care advice, and some actively calibrated their responses to take own control over when to be contacted by the nurses. A study in the same sample also demonstrate that the use of Interaktor reduced symptom burden, particularly concerning urinary-related symptoms and emotional functioning [28]. One explanation could be that the patients’ use of Interaktor enhance an active role in taking control over their own well-being and health. It is known that patients need and want to engage in active participation at different levels [38]. Patient participation is built upon relationships and shared knowledge [39], but this may be difficult to achieve today, as health care providers’ time with patients is reduced. Angel and Frederiksen [39] state in their review that a mutual relationship is difficult to achieve if a physical and temporal space is not established. Others report that to achieve patient participation, extended conversations are not required [40]. In face-to-face interviews in the same study sample, the patients using Interaktor described how the app facilitated and increased their involvement in care and that a mutual relationship was achieved between the patient and the health care providers, which was not so apparent in the control group [41]. More studies are required before conclusions can be drawn about patient outcomes, for example, on quality of life and clinical recovering. However, Interaktor apparently offers an interactivity with the health care providers that facilitates patients to feel secure, which might be a motive to high adherence of using the app.

### Methodological Considerations

The study has some methodological limitations. The patients who entered the study may have been more interested in using mHealth than nonparticipants, which might have impacted the findings. However, the participation rate, that is, 62%, is comparable with interventional clinical studies and is considered acceptable [42]. This study sample had a mean age of 69 years and thus was a cohort of older adults. In the literature, there have been discussions about the challenges older people can face with new technologies [43-45]. The lack of technical skills among older people and health care professionals has been described as hindering the implementation of information communication technology innovations [43,44]. Furthermore, lack of Internet access, problems with logging in, and unreliable wireless coverage have been described, which may decrease the participants’ accessibility and interest [45]. This was not apparent in this study, and there were very few technological problems described. Technological development is rapidly moving forward and doubts around older peoples’ interest and ability to use technological tools seem to be disappearing. In Sweden, 81% of the citizens are smartphone users, and it continues to rise [46]. Moreover, 58% of people over 65 years of age use a smartphone, and among those 75 years and older, 47% have a smartphone. The figures are similar in Germany [47]. Another reason for nonparticipation and dropout can be apprehension concerning cognitive accessibility or that the content is not user-friendly [22,48,49], but the patients in this study found the app to be user-friendly with relevant content, although it might not be so for all patients. Another strength of this study is the high adherence to daily reporting indicating that the use of mHealth is promising as an important tool in clinical care. Another limitation is that the interviews were not audiotaped, instead data collection was made by taking notes in a template following the interview guide. This could limit the trustworthiness of data because using notes taken by researchers may make the analysis to be based on already filtered content. However, 4 test interviews using the template showed that it was sufficient to take notes during these short interviews. Confirmability is attained as the research members had methodological experience with content analysis and different professional backgrounds.

### Conclusions

Patients with locally advanced prostate cancer adhere to, appreciate, and face few obstacles using an app for reporting and managing symptoms on a daily basis during radiotherapy. The Interaktor seems to consider patients’ different needs because it has several components that the patients can choose depending on their own needs. The patients felt secure when being monitored, and using the Interaktor increased their own reflections about their own well-being. The Interaktor seems to enable self-management and serves as a facilitator to attain person-centered care, although some adjustment and further development of the content will be beneficial for future use.

## Abbreviations

EBRT: external beam radiation therapy
mHealth: mobile health
N/A: not applicable
PROMs: patient-reported outcome measures
PIN: personal identification number
